# The rapid progress in COVID vaccine development and implementation

**DOI:** 10.1038/s41541-022-00442-8

**Published:** 2022-02-10

**Authors:** Alan D. T. Barrett, Richard W. Titball, Paul A. MacAry, Richard E. Rupp, Veronika von Messling, David H. Walker, Nicolas V. J. Fanget

**Affiliations:** 1 Sealy Institute for Vaccine Sciences and Department of Pathology, University of Texas Medical Branch, Galveston, TX USA; 2 College of Life and Environmental Sciences, University of Exeter, Devon, UK; 3 Department of Microbiology and Immunology, Yong Loo Lin School of Medicine, National University of Singapore (NUS), 5 Science Drive 2, Blk MD4, Level 3, Singapore, 117545 Singapore; 4 Department of Pediatrics, University of Texas Medical Branch, Galveston, TX USA; 5 Life Sciences Unit, Federal Ministry of Education and Research, Berlin, Germany; 6 Center for Biodefense and Emerging Infectious Diseases, University of Texas Medical Branch, Galveston, TX USA; 7 Nature Research, London, UK

**Keywords:** Vaccines, Public health, Drug discovery, Viral infection

January 2022 is the second anniversary of the identification of Coronavirus disease 2019 (COVID-19)^1^ caused by severe acute respiratory syndrome (SARS) coronavirus 2 (SARS-CoV-2). Scientifically, during the COVID pandemic, we have come a very long way in a very short period of time and demonstrated the power of twenty-first century science and technology when a pandemic situation catalyzed the adoption of novel vaccine technologies at record speed. Rapid sequencing of the virus genome allowed initial development to start in January 2020^2^ and we had our first authorized vaccines by December 2020^3^.

*NPJ Vaccines* has published over 65 papers on SARS-CoV-2 that cover the entire breadth of vaccinology from basic science to attitudes of the public to COVID vaccines. With this in mind, the editors of *NPJ Vaccines* have selected 17 articles that exemplify the rapid progress made with COVID vaccine development in the last 2 years.

Our first COVID paper described the outbreak of SARS-CoV-2 pneumonia in China with the first confirmed cases on December 29, 2019, and the urgent need for vaccines^4^. The scientific and medical community quickly realized that a vaccine would be essential for controlling the disease. Work started on developing inactivated, live attenuated, nucleic acid, subunit and vectored vaccines, and the various potential technologies and their advantages and drawbacks were summarized briefly in a Comment^4^. As laboratories around the world shifted to studying the virus, its biology and interactions with the immune system were also clarified.

SARS-CoV-2 is typical of many RNA viruses being enveloped with a major surface glycoprotein (in this case the Spike (S) protein) that is involved in binding to the cell receptor of the virus, angiotensin-converting enzyme 2 (ACE2), and is the major target for neutralizing antibodies. In particular, the receptor binding domain (RBD) of the S-protein is the target of the most potent neutralizing antibodies^5^. Importantly, immunization can achieve higher levels of antibody to the S-protein than natural exposure to the virus^6^. However, like many RNA viruses SARS-CoV-2 has a low-fidelity replication complex that allows the viral genome to mutate rapidly as it adapts to new conditions. Many SARS-CoV-2 laboratory isolates have been made in monkey kidney Vero cells. A very important study by Funnell et al. (2021)^7^ showed that Vero cell culture passaging of isolates must be undertaken carefully, as this can lead to the generation of virus variants with critical changes in the region of the S-protein furin cleavage site, affecting the use of such viruses in in vitro neutralization tests and in vivo challenge studies. Similarly, there are a number of different neutralization assays used to measure neutralizing antibodies and there is a need to standardize such assays using the World Health Organization standard that measures neutralization titers in International Units^8^. Mutations in the S-protein have proved to be particularly important for vaccine development, as they can result in changes in the interaction of the protein with both ACE2 and antibodies. The first major mutation identified in the S-protein was the D614G substitution, which became the dominant variant by June 2020. A range of studies reported in *NPJ Vaccines* have shown that antibodies to the Wuhan spike protein are able to neutralize a range of variants, including the Omicron variant, albeit with reduced potency towards some variants^9^, which is mostly due to mutations in the RBD of variants^5^. The reduced neutralizing activity of antibodies towards variants can, to some extent, be addressed using a third immunizing dose^10^.

Although vaccines depend on the native S-protein for inducing potent neutralizing antibody responses alongside T-cell responses, the presentation of the S-protein to the immune system differs substantially between the different vaccine platform technologies^11^. It is clear that differences in the presentation of the S-protein to the immune system can have a profound effect on the nature of the immune response^11^. This is elegantly illustrated in a study on the immune responses to a human adenovirus 26-vectored vaccine encoding modified forms of the S-protein^12^. A wide range of other approaches have been proposed, including using the anti-tuberculosis vaccine Bacille Calmette-Guérin (BCG), which has inherent immunostimulatory properties^13^. This might provide ways of re-programming the innate response to achieve single-dose protective immunity and could be of great value in both high- and low-income countries.

One of the intriguing features of the immune response induced by many coronavirus infections is the lack of a long-lived protective immune response, in particular the waning antibody responses. Bachmann et al. (2021)^14^ suggest this is due, in part, to the topography of SARS-CoV-2 virus, where S-protein is perpendicular to the surface of virions and embedded in a fluid membrane, such that neutralizing epitopes are loosely “floating.” Another feature of the S-protein is the N-linked glycosylation sites, which can mask epitopes. In the case of SARS-CoV-2, it was found that glycans masked epitopes on the S2 subunit domain of the S-protein, but not the S1 subunit domain, which includes the RBD^15^. These findings highlight the importance of understanding the basic biology of the virus to enable the development of effective vaccines.

Meanwhile vaccine development continued apace, and by February 2021 Kyriakidis et al. (2021)^16^ described 64 candidate vaccines, developed using different technologies (mRNA, replication-defective viral vector, virus-like particle, inactivated virus and protein subunit), as they entered phase III clinical trials. By May 2021 McDonald et al. (2021)^17^ were able to compile a systematic review and meta-analysis that compared reactogenicity, immunogenicity, and efficacy of 18 candidate vaccines based on studies in non-human primates and humans. Not surprisingly, the different vaccines varied in their abilities to induce antibodies (including neutralizing antibodies), T-cell responses, and their reactogenicity and efficacy.

Following the authorization or licensing of a vaccine, implementation becomes the critical issue, especially with respect to vaccine hesitancy. Kreps et al. (2021)^18^ investigated attitudes of the general public towards COVID-19 vaccination and identified that the public were confused in their understanding of the differences between “Emergency Use Authorization” and conventional “licensure.” The findings of studies such as this one have implications regarding public health strategies for implementation of many vaccines, not only COVID, to increase levels of vaccination in the general public. Another concern of the public is around the safety and efficacy of vaccines in special populations. For example, Low et al. (2021)^19^ undertook an important study that demonstrated codominant IgG and IgA expression with minimal vaccine mRNA in milk of lactating women, who received the Pfizer-BioNTech BNT162b2 and no adverse events in infants who breastfed from these vaccinees.

Finally, the COVID pandemic has identified the need for pandemic preparedness in the future for other pathogens and Monrad et al. (2021)^20^ discuss the important issues of how we could finance such activities moving forward.

We hope that you will find these papers interesting and informative, they are representative of the work we have published in *NPJ Vaccines*. We encourage you to look at these other equally interesting reports, which collectively provide an incomparable breadth of information and a resource for everyone interested in this field.
